# Chylothorax and Chylous-Like Diseases in Children: Clinical Management

**DOI:** 10.3389/fped.2019.00258

**Published:** 2019-06-27

**Authors:** Michael Hermon, Elias Tenner, Gudrun Burda, Wolfgang Strohmaier, Gerald Schlager, Johann Golej

**Affiliations:** ^1^Division of Neonatology, Pediatric Intensive Care and Neuropediatrics, Medical University of Vienna, Vienna, Austria; ^2^ENT-Department, Universitätsklinikum St. Pölten, Sankt Pölten, Austria; ^3^Institut für Molekulare Biowissenschaften, Karl Franzens University Graz, Graz, Austria

**Keywords:** chylothorax, chylous-like, pseudo-chylothrax, lymph, thoracic duct, children, therapy

## Abstract

**Background:** Chylothorax and chylous-like diseases are rare conditions and difficult to treat. But they may represent potentially life-threatening disorders and important causes of morbidity and prolonged hospitalization, especially in critically ill children. Conservative as well as surgical therapeutic management strategies are continuously performed at our institution, however the results have never been evaluated and no guidelines for treatment recommendations have been put into practice so far. The objective of this retrospective study was to present a comprehensive and substantial evaluation of all relevant demographic data from children with the chylothorax and chylous-like diseases and their clinical management.

**Methods:** We retrospectively analyzed data from all children with diagnoses of chylothorax and chylous-like diseases admitted to our pediatric intensive care unit between the years 1999 and 2012.

**Results:** Data of 34 patients were analyzed for this study. Gender distribution (M/F) was almost equal (19/15; 56%/44%). Thirty-one children (91%) developed chylothorax after surgery. Two children (6%) had idiopathic chylothorax and in one child (3%) congenital chylothorax was diagnosed. All study patients (*n* = 34; 100%) received MBF/MCT therapy. We were quite successful in treating 14 children who received only this therapy, with chest tube output dropping from 100 to 4.7%. But only 11 (32%) children received somatostatin and 7 (20%) children received beta-isodona. Different surgical interventions were performed in 6 patients (17%). All study patients received chest tubes to drain the pleural fluid and hence to relieve the chyle related symptoms.

**Conclusion:** A combination of different conservative therapies was successful in most of our patients. Prevention, early diagnosis and treatment of potential complications may further improve the success rate of conservative therapy especially in patients with postoperative chylothorax. In summary, appropriate therapy of this condition may be lengthy but can prevent significant morbidity and mortality.

## Background

Chylothorax is a rare accumulation of chylous lymphatic fluid in the thoracic cavity, which arises from a wide spectrum of causes and can be encountered in diverse clinical contexts (1). With an estimated prevalence of 1:15,000 deliveries and a postoperative risk of 0.2–2% in pediatric surgery, chylothorax poses an omnipotent challenge, especially in the treatment of critically ill children worldwide (1–3). Chylothorax is an important cause of morbidity and prolonged hospitalization in pediatric patients. Despite the low prevalence of chylothorax there are several classification schemes for this disease that describes the etiologies (4, 5). The anatomy and course of the thoracic lymphatic system can present with various differentiations. About 65% of the population has a “standard” anatomical route of the thoracic lymphatic system (4, 6). It is clear that chylothorax can occur after any surgery performed in the vicinity of the thoracic duct or its lymphatic tributaries, which together transport about 4 Liters of chyle every day (7). Depending on the level of chylous effusion, fluid accumulations can occur at any place in the thoracic cavity. Accumulation in the pleural space is typical but also chylopericardium and chylous ascites may result after injuring the chyle-transporting thoracic duct (8, 9). As effusions into the thoracic cavity lead to compression of the lungs and other intrathoracic structures, chylothorax can promote severe mechanical restrictions. Conservative as well as surgical therapeutic interventions are currently in use, but reports are scarce. Because of the rareness, the different etiology, and clinical presentation of chylothorax, single center studies may contribute to improve guidelines and facilitate treatment of these patients (10, 11).

The objective of this retrospective study was to present a comprehensive and substantial evaluation of all children treated with the diagnoses of chylothorax and chylous-like diseases at our institution. We generated the hypothesis that several combined conservative therapeutic measures might significantly reduce daily chest tube output in children with chylothorax. In non-responders, the surgical intervention might have been a successful treatment option.

## Methods

After approval of the ethics committee (Medical University of Vienna; Ethic Nr.- 1629/2013) patient records of all children admitted to our pediatric intensive care unit (PICU) at the Medical University of Vienna between 1999 and 2012 were screened for this study. A total number of 40 children were treated for the diagnoses chylothorax or chylous-like diseases at PICU during this time period and were included in this retrospective study. Because of the retrospective character of this study, informed consent was waived and the study was performed in accordance with the Declaration of Helsinki.

All study patients met the following inclusion criteria: children with chylothorax with an age that ranged from newborns up to < 18 years. Exclusion criteria were: Age > 18 years, transfer to other institutions or hospitals, missing or deficient documentation of clinical databases.

Chylothorax was suspected when chest x-ray demonstrated fluid accumulation in the pleural space, or when drained pleural fluid appeared to be turbid and milky. When biochemical analysis of fluid revealed a triglyceride level above 110 mg/dl, diagnosis was confirmed. The diagnosis of chylous-like disease (pseudo-chylothorax) was based on the biochemical (triglyceride level below 110 mg/dl; elevated cholesterol above 200 mg/dl in pleural fluid) and microscopic analysis of the pleural fluid (12).

The following data were extracted from patient files: age (days), gender, PICU stay (days), diagnosis at admission. Chylothorax etiology (congenital, postoperative, lymphangiomatosis, idiopathic, other). Chylothorax localization (right/left/bilateral), chest tube localization (right/left/bilateral). Type of thoracic surgery. Chest tube duration (days), drainage amount (ml/kgBW/day). Type of Nutrition: Milumil Basic-F (MBF) (Milupa GmbH; Puch bei Hallein, Austria), medium-chain triglicerides (MCT), duration of nutrition (days). Therapy with Somatostatin (Yes/No). Therapeutic Pleurodesis with beta-isadonna (BID)/Iodopovidone (Yes/No). Laboratory analysis (Triglycerides and Cholesterol in pleural fluid and blood). Surgical Interventions e.g., ligation, pleurectomy, percutaneous transluminal angioplasty (PTA), Thoracic Duct Embolization (TDE) (Yes/No).

Data Documentation was performed in a dedicated database (Microsoft Excel, Microsoft Austria GmbH, Vienna). Data analysis was performed using IBM SPPS Statistics Version 21.0 (IBM Germany GmbH, Ehningen). For statistical analysis Chi-square test or Fisher's exact test and independent-samples Kruskal-Wallis test were used. Results were accepted as statistically significant at *p* < 0.05. Data are presented as median and interquartile range or percentage.

## Results

Hospital records of all children with chylothorax admitted to our PICU between 1999 and 2012 were reviewed. The diagnosis was detected in 40 children, but 6 patients (15%) had to be excluded, due to incomplete documentation or transfer to other hospitals. Data of 34 patients were analyzed for this study. Table 1 shows demographic data of study patients. Gender distribution (M/F) was almost equal (19/15; 56%/44%).

**Table 1:** Demographic data.

**Patients (N)**	34
Gender (m/f)	19/15
**Age** (days) (Median/IQR)	95 (10/241)
**Weight (kg)** (Median/IQR)	4.2 (3.3/6.6)
**PICU stay** (days) (Median/IQR)	14 (7/78)
**Etiology**	
Cardiac Surgery	28
Other Surgery	3
Idiopathic/Congenital	3
**Physiology**	
Biventricular	32
Univentricular	2
**Heterotaxie**	None
**Syndrome**	
Down-Sydrome	8
Other (Cat-eye, Dysmorphic feature)	2
**Side**	
Right	9
Left	9
Bilateral	14
Chylopericard	2
**Survival**	34

Thirty one children (91%) developed chylothorax after surgery. Two children (6%) had idiopathic chylothorax and one child (3%) was diagnosed with congenital chylothorax.

Within this postoperative group, 28 children had cardiac surgery, two children had congenital diaphragmatic hernia (CDH) and one child had liver surgery. The 28 children who developed chylothorax after cardiac surgery were divided into three categories: corrective surgery, palliative surgery, and other surgery.

Laboratory examination of pleural fluid was performed in 27 children and a total number of 21 children fulfilled the criteria for diagnosis. In seven children, pleural fluid analysis was not performed or at least not documented in the database. In six children, the diagnosis of chylous-like disease (pseudochylothorax) was confirmed through a comparison of pleural and blood triglycerides and cholesterol values, obviously obtaining higher values in blood circulation than in pleural fluid.

The side of effusion was noted for correlation between localization of chylothorax and its underlying etiology and the type of surgery (see Table 2).

**Table 2:** Correlation between type of surgery and chylothorax localization.

**Type of surgery**	**Side Right**	**Left**	**Bilateral**	**Chylopericard**
Corrective N (%)	5(26)	4(21)	9(48)	1(5)
Palliative	3(38)	2(25)	3(38)	0
Other	1(25)	2(50)	0	1(25)
Total	9(29)	8(26)	12(39)	2(6)

**Figure 1:**
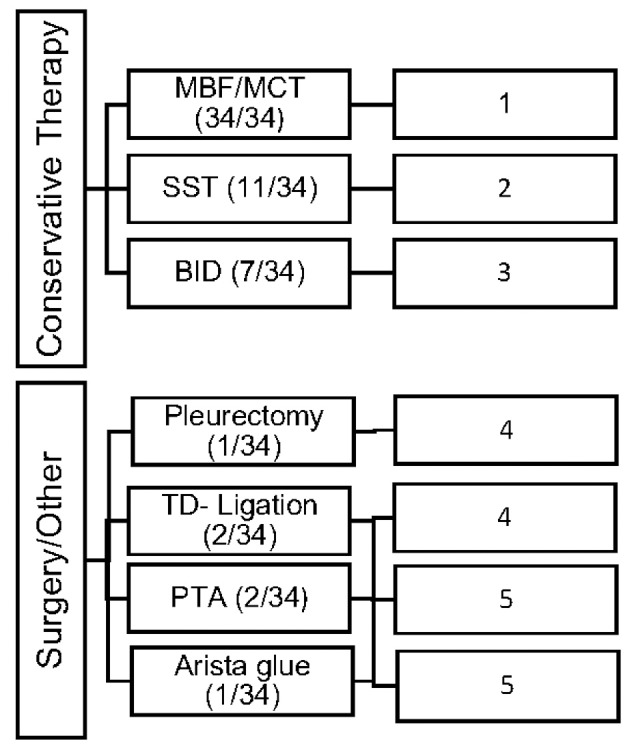
Overview of the frequency of therapeutic interventions divided into 5 categories of therapy (Type 1–5).

Conservative as well as invasive therapeutic interventions (surgical) have been practiced continuously in our patients. In many cases a combination of different management strategies was necessary to decrease the amount of effusion. The different therapies were categorized in 5 different types (Number 1–5; see Figure 1).

All study patients (*n* = 34; 100%) received MBF/MCT therapy, while some received somatostatin (*n* = 11; 32%) and BID (*n* = 7; 20%). Different surgical interventions were performed in 6 patients (17%).

All study patients received chest tubes to drain the pleural fluid and hence relieve the chyle related symptoms. Chest tube outputs for all patients (ml/kgBW/day) before starting any kind of therapy and before removing the chest tubes are shown in Table 3.

**Table 3:** Chest tube output.

**Patients ID**	**Chest tube Output before start of Therapy (ml/kgBW/day)**	**Chest tube Output before chest tubes were removed (ml/kgBW/day)**	**Therapy time (days)**
1	21	2	4
2	No data	No data	No data
3	53	142	11
4	93	5	142
5	12	2	3
6	50	No data	
7	28	0	5
8	113	4	38
9	19	2	3
10	104	20	77
11	12	2	3
12	52	0	49
13	40	4	33
14	18	1	4
15	101	No data	No data
16	52	3	5
17	No data	No data	No data
18	No data	No data	No data
19	No data	No data	No data
20	48	7	20
21	25	2	7
22	61	26	8
23	25	3	4
24	136	9	70
25	33	23	11
26	No data	No data	No data
27	No data	No data	No data
28	No data	No data	No data
29	52	26	7
30	36	8	5
31	42	9	28
32	18	2	5
33	No data	No data	No data
34	No data	No data	No data

All study patients received also nutritional modifications to MBF/MCT, while 14 children received only MBF/MCT and the remaining patients (20 children) received MBF/MCT in combination with the 4 other types of therapy. In the group of 14 patients who received only MBF the median therapy time was 5 (4–8.5) days. The median chest tube output before starting MBF/MCT therapy was 24.5(17.7–45) ml/kgBW/day and dropped to 1.1(0–2.8) ml/kgBW/day just before or on the day chest tubes were removed.

**Figure 2:**
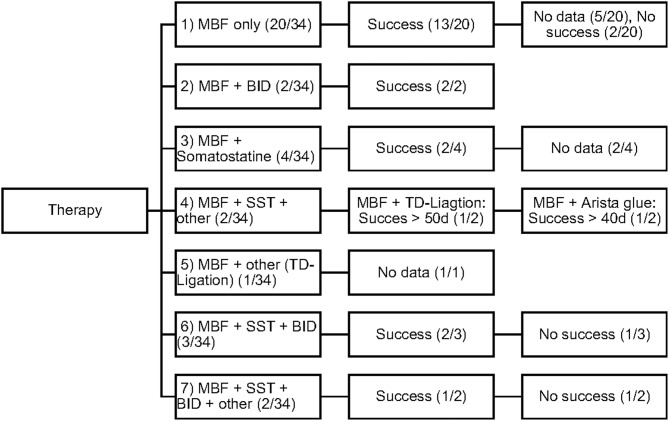
Therapeutic combinations in study patients.

The efficacy of each therapeutic procedure was treated as a binary variable: success or failure. Each therapy was considered to be successful when the decrease in daily chest tube outputs after 3–5 days of therapy was > 60% or even when, in some patients, total cessation of chyle flow was achieved. Figure 2 displays the different therapeutic combinations as well as their efficacy.

All patients survived PICU-stay and were transferred to the normal ward in the children's hospital.

## Discussion

Chylothorax is a rare condition and difficult to treat. Despite its rareness, it can represent a potentially life-threatening disorder in critically ill children.

The objective of our study was to present a comprehensive and substantial evaluation of the various treatment strategies that have been practiced over the past years in children with chylothorax in our hospital. We therefore systematically reviewed the medical records of our patients with this rare disease and focused our investigations on underlying etiology, diagnostic pathway, and clinical survey of each individual case. Demographic data are listed in Table 1. Distribution of gender (m/f) was almost equal (56%/44%) without any gender influence on the clinical course. This findings correspond with other reports (14–16). Chylothorax etiology is just as heterogenetic as described in the literature. Most patients in this study (28, or 82%) developed chylothorax after cardiothoracic surgery. Although we did not estimate the overall incidence of postoperative chylothorax in our hospital due to inaccessible or missing data, published literature estimated the incidence in children at 2–4.5% (17). The incidence of chylothorax in children is lower than in adults (4). In most cases traumatic chylothorax occurs 2–4 days after trauma or surgery. Those patients usually had already chest tubes, which suddenly start to present chylous fluid with oyster white color. Non-traumatic chylothorax and chylous-like disease are usually diagnosed by chest x-ray, CT-scan or sonography of the lungs because other diseases are concomitantly present (18). Bilateral effusions were most frequently observed and diagnosed. With regards to cardiac surgery, previous studies have shown that extrapericardial procedures, such as Blalock-Taussig shunt placement, aortic coarctation repair, patent ductus arteriosus ligations, and Glenn procedures, are mostly responsible for the formation of chylothoraxes (17, 19–21). Among all study patients, 32 children had biventricular and only two children univentricular physiology. None of the patients had hetorotaxie. Description of the diagnostic pathway has been an important part of our study. In six children the peak values for triglycerides in the pleural fluid were below 110 mg/dl, indicating the diagnosis chylous-like disease (pseudo-chylothorax) for those children. This group of patients were treated similarly to the patients with chylothorax. Another crucial point in our study was to present a therapeutic overview about all the different management strategies that have been performed. In 14 patients a combination of different therapies was necessary to decrease the amount of effusion above 60%. Figure 2 provides an overview about the frequency of therapeutic interventions practiced in this study. We hereby differentiated between conservative, surgical, and other-therapies. All study patients received chest tubes to drain the pleural fluid to relieve respiratory symptoms. It is well known that feeding restricted to medium- or short-chained triglycerides results in reduced lymph flow in the TD and may enhance spontaneous healing (21). While this conservative approach alone could not resolve the chylothorax in all cases, we were quite successful in the treatment of 14 children who received only this therapy. Median chest tube output before starting MBF/MCT therapy was 24.5 (17.7–45) ml/kgBW/day and dropped to 1.1(0–2.8) ml/kgBW/day, just before or on the day chest tubes were removed. This equates to a drop from 100 to 4.7% (0–22.3). Previous studies have shown that any type of enteral feeding, even with clear fluids, can cause an increase in the TD-flow (22). Therefore, some authors recommend chest tube drainage, withholding of oral feeds, and providing total parenteral nutrition for the optimum management of chyle leaks (21). The use of iodopovidone (BID) for pleurodesis is also considered as a well-established therapy, which was shown to be effective in several studies (23–25). In our study, this technique was performed on seven children (see Figure 1), with two children receiving BID as the only therapeutic option beside the nutritional therapy and five children receiving BID in combination to other therapies. Cessation of chylous flow occurred in most patients between 3 and 15 days after the installation of BID. Chemical pleurodesis with “Arista glue” was performed in one patient, with a modest outcome. We did not use other agents for chemical pleurodesis, such as talc, although these have been widely used and well-established (26–28). Furthermore, high rates of efficacy were reported for the use of somatostatin in few reports, with the therapeutic effect usually occurring within the first 6 days of treatment (29–32). In our patients, somatostatin was applied in 11 children, with four children receiving somatostatin as the only therapeutic add-on to nutritional therapy. Three children received somatostatin in combination to BID and four children either had to undergo surgery or be treated with somatostatin combined with other management strategies. In general, surgical interventions are required for intractable cases when chylothorax remains resistant despite prolonged drainage and conservative management (17, 21). Because of the small number of patients (six children) with partially incomplete documentation, it is difficult to determine the therapeutic role of surgical management in our unit.

Due to the retrospective character of our study, there are few limitations which is important to mention: first, a complete documentation of parameters is necessary for a coherent and sound interpretation. Relevant data, such as the daily chest tube flow, was unfortunately only partially/poorly documented. Second, one has to consider that the clinical survey of chest tube flow is also influenced by several factors that have not been considered in our methods. Increased systemic or venous pressure, as well as other co-morbities such as pre-existing heart insufficiency or vascular disease, might have affected the course of chest tube flow and contributed to prolonged hospitalization in some cases.

## Conclusion

**Figure 3:**
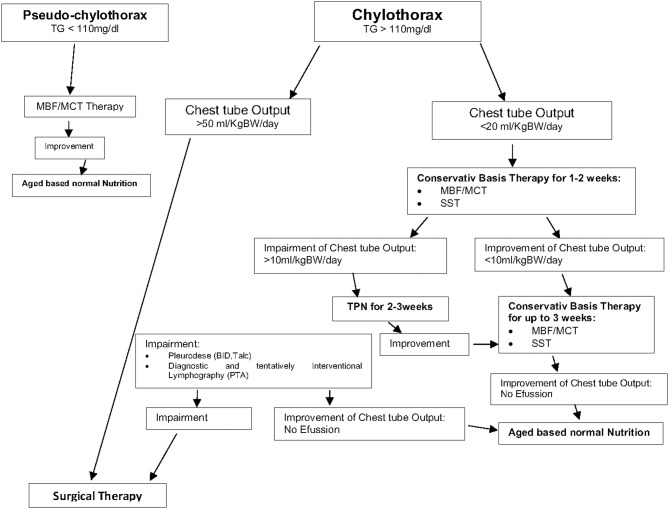
Therapy of chylothorax and chylous like diseases—Clinical Algorithm modified (13).

We noted that a combination of different conservative therapies such as nutritional modification (MBF) and somatostatin was successful in most of our patients. Therefore, conservative therapy is recommended as the preferred therapy in the presence of chylothorax and chylus-like diseases [see Figure 3; Clinical Algorithm modified; (13)]. Surgical therapy may be an alternative approach that can be used when conservative therapy has failed. Early diagnosis and treatment of potential complications (high central venous pressure > 15 mmHg, heart failure) may further improve the success rate of conservative therapy especially in patients with postoperative chylothorax. In summary, appropriate therapy of this condition may be lengthy but can prevent significant morbidity and mortality. Therefore, a suitable protocol is needed to define uniform diagnosis criteria and treatment modalities for this special medical condition.

## Data Availability

The datasets used and/or analyzed during the current study are available from the corresponding author on reasonable request.

## Ethics Statement

All procedures performed in studies involving human participants were in accordance with the ethical standards of the institutional and/or national research committee and with the 1964 Helsinki declaration and its later amendments, or comparable ethical standards. For this type of study (retrospective study) informed consent is not required. This study was performed with the approval of the ethics committee (Medical University of Vienna; Ethic Nr.-1629/2013).

## Author Contributions

MH conceived the study and participated in the design and execution of the study, the analysis of data, and writing of the manuscript. ET participated in the design and execution of the study, and performed data collection, statistical analysis, interpretation of the data, and writing of part of the manuscript. GB performed data collection. WS participated in the interpretation of results and critical editing and writing of the manuscript. GS performed statistical analysis, interpretation of the data. JG supervised the study and is program director. All authors read and approved the final manuscript.

### Conflict of Interest Statement

The authors declare that the research was conducted in the absence of any commercial or financial relationships that could be construed as a potential conflict of interest.

## Abbreviations

CDH: Congenital diaphragmatic hernia
PICU: Pediatric Intensive Care Unit
TD: Thoracic Duct
TDE: Thoracic Duct Embolization
PTA: percutaneous transluminal angioplasty
PP-Shunt: Pleuroperitoneal Shunt
TG: Triglycerides
Chol: Cholesterine
MCT: Medium-chain triglicerides
MBF: Milumil basic F
SST: Somatostatine
BID: Beta-isodona.
